# Lectures on Scarlet Fever

**Published:** 1851-03

**Authors:** Caspar Morris

**Affiliations:** Late Lecturer on Practical Medicine in the Philadelphia Medical Institute


### MEDICAL EXAMINER,

AND

RECORD OF MEDICAL SCIENCE.

NEW SERIES.—NO. LXXV.—MARCH, 1851.

---

### ORIGINAL COMMUNICATIONS.

---

*Lectures on Scarlet Fever.* By Caspar Morris, M. D., late Lecturer on Practical Medicine in the Philadelphia Medical Institute.

### Lecture II.

The malignant being by far the most serious form of the disease, not only from the severity of the features and gravity of the results in individual cases, but from the fact that its epidemic visitations are more wide-spread and frequent than those of the milder forms, you will, I trust, pardon me, if I dwell somewhat at length upon the description of it. To the account of the disease as it appeared in London in 1747–8, just quoted from the accurate and conscientious Fothergill, allow me to add that of our own first medical writer of distinction, Dr. Benjamin Rush, who in his Medical Observations and Enquiries, thus describes the occurrence of this form of the disease in the year 1783, and 1784, in this city. "In most of the patients who were affected by it, it came on with a chilliness and a sickness of the stomach, or a vomiting, which last was so invariably present that it was with me a pathognomonic sign of the disease. The swelling of the throat was in some instances so great, as to produce a difficulty of speaking, swallowing and breathing. In a few instances, the speech was accompanied by a squeaking voice, resembling that which attends the cynanche trachealis. The ulcers on the tonsils were deep, and covered with *white*, and in some instances, with *black* sloughs. In several cases there was a discharge of a thick mucus from the nose, from the beginning; but it oftener occurred on the decline of the disease, which not unfrequently happened on the fifth day. Sometimes the subsiding of the swelling of the throat was followed by a swelling behind the ears."

"An eruption on the skin generally attended the symptoms, which have been described. But this symptom appeared with considerable variety. In some people it preceded, and in others it followed the ulcers and swelling of the throat; in some it appeared only on the outside of the throat, and on the breast; in others it appeared chiefly on the limbs; in a few it disappeared on the second or third day of the disease, and never returned afterwards. I saw two cases of eruption without a single symptom of sore throat."

"The fever which accompanied the disease, was generally the the typhus mitior of Dr. Cullen. In a few cases it assumed symptoms of great malignancy. The disease frequently went off with a swelling of the hands and feet. I saw one instance in which the swelling was absent, but the patient complained of very acute pains in the limbs, resembling those of rheumatism."

"In two cases which terminated fatally there were large abscesses; the one on the outside, the other on the inside of the throat. The first of these cases was accompanied by troublesome sores on the ends of the fingers. One of these patients lived twenty-eight, and the other above thirty days, and both appeared to die from the discharge which followed the opening of the abscesses."

"Between the degrees of the disease which I have described, there were many intermediate degrees of indisposition which belonged to this disease."

"I saw in several cases a discharge from behind the ears and from the nose, with a slight eruption, and no sore throat; all three patients were able to sit up, and walk about. I saw one instance of a discharge from the inside of one of the ears in a child, who had ulcers in the throat, and the squeaking of voice. In some, a pain in the jaw, with swellings behind the ears, and a slight fever, constituted the whole disease. In one case the disease came on with a coma, and in several patients it went off with this symptom. A few instances occurred of adults who walked about, and even transacted business until a few hours before they died."

"The intermitting fever, which made its appearance in August, was not lost during the month of September, (the time at which the scarlet fever became prevalent.) It continued to prevail, but with several peculiar symptoms. In many persons it was accompanied by an eruption on the skin and a swelling of the hands and feet. In some it was attended by a sore throat and pains behind the ears. Indeed, such was the predominance of the scarlatina anginosa, that many hundred people complained of sore throat without any other symptom of indisposition. The slightest occasional or exciting cause, particularly cold, seldom failed of producing the disease."

"The epidemic prevailed in Philadelphia, from September throughout the winter. In the Spring it disappeared, but spread afterwards through the neighboring States of New Jersey, Delaware and Maryland."

You will here observe, that while the prevailing type of the epidemic thus described, is of that severe character which brings it under the form of the disease which now claims our notice, the occurrence simultaneously of cases of a milder degree, prove their dependence on the same cause. Were it proper to burden your memory or exhaust your attention by swelling these quotations, I might add to them similar descriptions of the disease given by distinguished writers upon medicine in each succeeding generation. But as you are, I trust, prepared to recognize it, should you ever be called upon to combat its malign influence, we will pass on to the consideration of the last of the four classes or groups of cases, into which I have proposed to distribute the disease.

While the *anginose* and *malignant* are the predominating types during every severe epidemic prevalence of the disease, and the *simple* form that which is generally found in sporadic cases, those which I am now to describe are found associated with the two former classes; occurring at the same time, and either in the same households, or in such circumstances as to prove their dependence on the same cause. The very term *Irregular*, by which I have designated this division, will at once convey to your minds, the suggestion that it must be impossible to give any general description which would embrace all the varieties included under that term. I shall therefore be compelled to enter into the detail of individual cases. By far the largest number owe their peculiar features to that condition of the nervous system which gives rise, in other febrile diseases also, to that collection of symptoms which marks what are erroneously called *congestive* cases. The vital forces are overwhelmed by the intensity of the morbid impression, and the patient dies without time being afforded for the disease to develop its essential features; or, if the duration of the case be more prolonged, so extreme is the force of the cause of disease, or so inadequate the power of resistance, that the development is only partially accomplished. The suddenness of the invasion has been noticed already as one of the peculiarities of scarlet fever. These cases are marked especially by this sign. I have known an infant, which had left home, apparently in perfect health, for its usual morning airing, brought back within an hour, with stupor and general muscular relaxation, cold surface, feeble pulse and total insensibility; no remedies that could be applied, produced any reaction, and it died within twelve hours. Two other children in the same family were seized within a few days, by scarlet fever, which manifested the usual signs, and ran a regular course, and thus placed beyond cavil the true nature of the case which preceded them. In another instance, while visiting a lady laboring under a chronic malady, my attention was drawn by the mother to the unusually healthful condition of an only child of two years old, gamboling on the floor. Within twelve hours, I was called to see it lying comatose and convulsed and within twelve hours more it was dead. A maculated appearance of the skin, and the known prevalence of scarlet fever in the vicinity, were here the only grounds for assuming this to be the nature of the disease, as there were no other children in the house, to be subjected to the poison. Dr. Rush, you will remember, reports "a few instances of adults, who walked about, and even transacted business, until a few hours before they died."

It is not many years since a Judge in one of our Courts was seized with nausea while on the bench, and retired to his home, where for two days he remained, scarcely willing to admit himself to be sick, and reluctant to confine himself to his chamber, though the rapid, feeble pulse, and an imperfect eruption, too plainly indicated the nature of the affection; and on the third day he died, while in the act of shaving himself.

Dr. George Gregory, in his lectures on Eruptive Fevers, delivered at St. Thomas' Hospital, London, reports the observation of cases of a somewhat similar kind. He says, "In some extreme cases, the nervous system shall be so completely depressed and subdued by the virulence of the miasm, and the mass of the blood so thoroughly poisoned and disorganized by it, that all the ordinary appearances of scarlet fever are masked; petechiæ, coma and a sloughy state of the throat alone appear. Life rapidly yields under such an attack." In confirmation of this assertion, he reports as having passed under his own notice, the case of a family, in which "a mother and two grown up daughters died. In each of the three cases the nervous system was utterly prostrated, and in a state of collapse. There was no violence, no delirium, no struggling for breath, but the pulse was small, the skin cold, and the whole system depressed by the intensity of the poison. Neither wine, brandy, nor capsicum could put any life into them. They sunk, one after another, without any attempt to rally. It was difficult to believe the, disease Scarlatina, but the eldest son took it in the usual form, and put the matter beyond doubt."

A lady, near the close of gestation, had the usual symptoms, which resulted from "taking cold" after fatigue and exposure, by which she was confined to the bed during two days, and from which she apparently quite recovered. After an interval of twenty-four hours, she was seized with a chill, followed by fever, with exceedingly rapid pulse and slight swelling and redness of the soft palate and uvula. I shall not detain you by the description of the treatment, which is irrelevant to the point now under observation, further than to remark that it was promptly administered and decidedly and actively antiphlogistic. There was never in the progress of the case, any great degree of internal swelling of the throat, and not the least about the neck externally; cough and dysphagia, with an entire inability to lie down, from the danger of suffocation, were the urgent symptoms. The heat of the skin was very great, but without any redness of the surface; the circulation continued exceedingly rapid throughout, and the countenance expressive of the greatest distress. The power of uttering sounds was lost, and there were some slight deposits of lymph on the uvula. This difficulty of swallowing, loss of voice, *rapid pulse* and great heat of skin, were the prominent symptoms, which continually increased in spite of the most energetic treatment, until she died on the fifth day of the disease, about twelve hours after having given birth to a living child. I was assisted in the treatment by three of the most eminent physicians of the city, none of whom suggested scarlet fever as the nature of the case. Examination of the body after death, exhibited, with sufficient clearness, the cause of these symptoms. A small abscess between the posterior walls of the pharynx and the vertebræ had caused the difficulty of swallowing, while minute deposits of pus beneath the mucous membrane on the arytenoid and cricoid cartilages, destroyed the voice. These lesions could not, however, have caused death so promptly, nor would they have given rise to the excessive rapidity of pulse, and heat of skin. Within a week after her death, two well marked and violent cases of scarlet fever, with severe anginose symptoms, occurred in the same house. One in the person of the nurse, who had waited upon the case I have thus described, and the other in that of a child.

In another instance, an infant, endowed with great vigor of constitution, was taken ill with febrile symptoms accompanied by slight difficulty of deglutition. No appearance of disease of the fauces was manifest to the most careful examination, beyond a very slight redness of the edge of the half-arches. Soon the voice became enfeebled, and the respiration impeded, but without cough. The pulse was very rapid, the skin hot and dry, but bloodless, presenting a waxlike paleness. The glands about the base of the jaw became swollen, the cellular tissues of the neck infiltrated with serum: the difficulty of swallowing increased, the voice became stridulous. These symptoms grew progressively worse till the child died, exhausted by the violence of the febrile reaction. Inspection of the body after death exhibited a superficial ulceration of the mucous membrane lining the larynx and upper part of the trachea, which had given rise to the glandular swelling and œdema. The rapidity of the pulse, heat of skin and peculiar condition of the neck, all combine to induce me to class this case with scarlet fever without hesitation. I have no doubt that other cases, well developed, would have followed, had there been children in the family. Many other similar cases have fallen under my notice, which have been so very evidently connected with this disease, as to forbid a doubt on the subject.

The course of the disease in the *Malignant* and *Irregular* forms, cannot be reduced to the regularity which marks the *Simple* and *Anginose*. In the simple uncomplicated form, the fever subsides from the 5th to the 7th day; and in favorable cases of the anginose variety, the primary lesions disappear with it, and convalescence is fairly established. When the ulceration of the throat and posterior nares is more severe, the primary fever is, on the contrary, merged insensibly into the fever of irritation produced by these lesions, and the case may run an uninterrupted course of many weeks; while in the malignant form, the patient may either sink into a comatose condition and die from the first force of the disease between the 3d and 9th day, or, if there be reactive force sufficient to carry the case over these periods, there still remains the irritation resulting from sloughing ulcers of the throat, abscesses beneath the jaws, and ulceration of the nares and ears, to be added to the depressing influence of the primary disease; and not unfrequently acute meningitis supervenes, and causes death with the usual symptoms of that disease superadded to those already described. Croup is also an occasional complication, either caused by the extension of the deposits already described as frequently occurring on the tonsils, or by the active inflammation of the larynx without any exudation. I have seen it in one instance prove fatal when the anginose symptoms had been very slight indeed, and where the most careful investigation could not detect the smallest exudation in the fauces. In the anginose malignant forms, the extension of the disease to the larynx and trachea is by no means uncommon, and I believe more careful observation will confirm the suspicion I entertain, that the disease known as Croup, which begins with inflammation of the tonsils and exudation of lymph—gradually extending to the larynx—is one of the forms of the disease now under consideration. To this conclusion I am led by having observed that such cases are particularly prevalent at the times when we know the scarlet fever influence to be most active, and from their evident dependence on some other cause than the mere vicissitudes of temperature which give rise to the inflammatory croup. The rapidity of the circulation, the tendency to gangrene of the throat, the general similarity of all the symptoms to those cases of undoubted scarlet fever in which the disease extends to the larynx, and the frequent occurrence of several cases in immediate succession in a family, all tend to the confirmation of this impression.

There has been some diversity of opinion as to the *cause* of scarlet fever. That it depends on some unknown agency, producing what is known as epidemic influence, no one denies. In what this influence consists it is impossible to determine, with our present limited knowledge and means of observation. Telluric emanations, sidereal influences, animalculæ and fungi, have all been invoked in their turn. It were vain to attempt to decide between these rival claims; a more important question demands our attention, and should be carefully examined. Is it ever propagated by contagion? The evidence in the affirmative is so positive that I cannot admit a doubt; so many well marked cases have passed under my own notice, and so many more are reported on authority quite beyond question, that I see not how any one can hesitate. It is asserted by those who disbelieve the contagiousness of the disease, that the instances in which single cases occur in a large family or school, without extension to others, even where no precautionary measures are adopted, are sufficiently numerous to disprove the influence of contagion; which, if characteristic of the disease, must operate equally at all times where it meets with subjects not protected from its attacks; and they aver that its extension, when this does take place, may be ascribed with greater propriety to a general exposure to the epidemic influence, the operation of which is acknowledged by all, than to contagion. Those who thus reason, however, overlook the fact, that even the diseases which all confess to be possessed of a power of propagating themselves are liable to the same uncertainty. I have, more than once known a single case of small pox to occur in a large family, without spreading, though no precautionary measures were employed; and what physician is there who has not been annoyed by the necessity for repeated insertions of vaccine virus, the susceptibility to the impression either wholly absent, or too feeble to be kindled into activity. The question of contagion is not one of merely speculative interest. Its practical bearings are highly important. Allow me, therefore, to mention some of the facts which prove it. I had a child under my care with scarlet fever of a very severe grade. The aunt came to the city from a section of country in which there was no disease prevalent, and took the complaint. A gentleman died of scarlet fever; a relative who had been alienated from him for many years visited the body, and died within a fortnight of the same disease. Those engaged in nursing cases of scarlet fever, and often all the adult members of a family in which it is present, are liable to sore throat and fever, without any eruption. The wife of a medical friend of mine, who was aiding in the care of the children of a relative, was seized with this modified affection, and communicated scarlet fever in all its integrity to her own children. It frequently happens that children who have had the disease absent from home, communicate it to others on their return during convalescence. This brings naturally to our notice another question. Can the principle of contagion attach itself to the clothing or persons of those who are not themselves affected by it, or the disease be conveyed or communicated by them? My own observation leads me to deny the possibility of the communication by mere transient intercourse. I have never, in long continued and extended practice, known the disease to occur in a family which I was visiting for other complaints, during my attendance, nor have I ever heard of an authenticated instance in which this has occurred. I have, however, known an instance in which a family had abandoned their dwelling immediately after the occurrence of two fatal cases.

The house was left uninhabited during several months; when a family, from a distant city, in which the disease was not prevailing, took possession of it, and the furniture which had been allowed to remain. Within two weeks the disease made its appearance in the family, and proved fatal to several children. Dr. Percival, of Dublin, than whom we can have no higher authority, in writing to Dr. J. Mason Good, says, "*Cynanche Tonsillaris* and *Maligna*, I consider with you a species of Roseola. (This is the term applied to scarlet fever by Dr. Good.) All have been produced by the same specific contagion, which in one instance was imported here from England in a Pandora's box, containing plumed soldiers, which had served to beguile the convalescent hours of a young family, and were sent by them as a present to their quondam playmates in this capital."

The late professor Hosack of New York, in supporting the doctrine of its contagious character, mentions the instance of a family who deserted their house after a fatal attack of scarlet fever. It was thoroughly cleansed and whitewashed, and the family remained absent six weeks. Immediately on their return, the disease reappeared. There is one source of fallacy in this case. The period of *incubation*, as it is termed, the time which elapses between the exposure to the sources of the disease and its development, during which the cause is supposed to lie latent in the system, but preparing to exhibit its characteristic features, is more uncertain in this, than any other disease of the same class. It is therefore not impossible that the germs of the disease may have been imbibed before leaving the house, either directly from the same source as the first case, or mediately through it. No such suspicion throws a shade on the instance I have mentioned before. The following cases will prove as well the contagious character of scarlet fever, as the uncertain duration of this latent period. A lady came from a section of country wholly exempt from this disease, to assist in nursing the child of a brother. Within forty-eight hours after her arrival, she was herself seized with a well marked attack. This is the shortest interval between exposure and sickening I have seen or read of. The longest I have known, was twenty-eight days. This case was equally well marked. A gentlemen designing to spend the summer in Europe, had engaged passage for himself and family to sail on the 1st May. One of his three children was seized with scarlet fever on the 1st day of March. Desiring that all should have the disease, and be quite recovered before leaving, no interruption of intercourse with the other children was attempted; the two children who were well were allowed free access to the room in which the sick one lay. The second seizure occurred on the 16th of March, and the third not until the 28th. Dr. Maton reports a case in which twenty-six days intervened. From eight to fourteen days is however the usual interval; and though occasionally several cases may occur simultaneously in a large family or school, it is much more common that one should be first taken sick, and another after an interval of greater or less duration, rarely beyond ten days; and then the other members of the family or household, who have not previously had the disease, are taken sick at about the same time, or in more rapid succession; while the adults are all more or less brought under the influence of the disease, with sore throat and fever. This mode of invasion by successive cases is almost uniform, and is certainly more easily accounted for, on the presumption of contagion, than in any other manner.

The attempt has been made to impart the disease by inoculation, but without success. This was to have been anticipated, as there is no analogy between this disease and variola, which would lead to the presumption it could be thus communicated. Whether introduced by contagion, or originating in some unknown atmospheric changes, scarlet fever is very often *epidemic.* In this respect it manifests a peculiarity as decided, as in other points of character. It is often bounded by very narrow limits. Not only will one part of a large city be exempt while it prevails in another, but I have known it confined to a single block of houses, while all the neighboring squares were healthy. Sporadic cases are frequently occurring, but these are always mild, and generally of the simple form, or with slight anginose symptoms. It often prevails in this mild manner, or entirely disappears for several years, until its malignity is almost forgotten, or may be even doubted. This was decidedly the case in our city for many years before 1830. Dr. Emerson says: "It is a remarkable fact, that in the twenty-one years from 1807 to 1827, inclusive, the total mortality from scarlet fever was only

102 out of 53,000 deaths. Doubtless some cases were reported in the bills of mortality under the vague title of sore throat, of which the amount during the whole period referred to, was 355; but with such additions, the proportional mortality from scarlet fever, would be trifling compared with its ravages in more recent years. *Only one death was reported in* 1827, *not one in* 1828. In 1829 there were 9, in 1830, there were 40. Since the last named year, the mortality from this source has very much increased, as will be apparent from the number of deaths reported in the subsequent ten years, viz.

| | | | |
|---|---|---|---|
| 1821, | 200. | 1836, | 240. |
| 1822, | 307. | 1837, | 205, |
| 1833, | 61. | 1838, | 134. |
| 1834, | 83. | 1839, | 225. |
| 1835, | 205. | 1840, | 244. |

Making a total mortality from this cause in ten years, of no less than two thousand and fonr cases." Dr. Emerson's conjecture that some scarlet fever cases were included in the 355 deaths from sore throat, is undoubtedly correct, as I know there was an epidemic angina during the period embraced in those returns. It was in the year 1828, in which only one death from scarlet fever is reported, I received an appointment in the Dispensary; and the district allotted to my care was that in which the epidemic, which began in 1829, and increased so fearfully till the year 1832, first exhibited its ravages. The contrast afforded by the comparison of the three years, 1827, 28, 29, with the three which followed, exhibits the point I wish to illustrate. So remarkable is this feature of the destiny of scarlet fever, that repeatedly it has been described as though it were a new disease—a whole generation of medical practitioners passing without the appearance of an epidemic.

In the year 1831, a highly respectable phyiscian, very extensively engaged in practice, told me he had never seen a fatal case of scarlet fever; his testimony thus corresponds with that of the bills of mortality for the period during which he had pursued his professional career. There is no fixed term of duration of these epidemic periods. In some instances a grand epidemic cycle appears, composed of series of minor epidemics. Thus, for instance, the table furnished by Dr. Emerson through the entire ten years, may be considered one epidemic period, in contrast with the twenty which preceded, there are two marked interruptions to the otherwise regular average mortality. Though, as I have before remarked, the epidemic visitations of this disease, generally partake of the typhous or malignant character, it occasionally happens that one may assume the sthenic form, and be attended with active inflammatory complications. In this respect the history of the past is instructive, as we shall find when we come to consider the treatment appropriate to the disease. Neither season of year, nor temperature appear to exert any influence over it. By some writers the winter, and by others the summer, is assigned as the period especially favorable to its diffusion. The worst epidemics I have seen, have been in the changing weather of the spring and fall.

(To be continued.)

